# Supplementary material for: How are people coping with working from home during the COVID-19 pandemic?: Experiences from the Netherlands and South Korea
Source: PLoS One. 2024 Apr 18;19(4):e0301351. doi: 10.1371/journal.pone.0301351 (PMC11025775; doi:10.1371/journal.pone.0301351)
Supplement: S1 Appendix — (DOCX) [file pone.0301351.s001.docx]

**Supporting information**

**S1 Appendix: Measuring and coding variables**

S1.1 Table. Outcome Variables

| **Type of variable** | **Coding** | **Data acquisition** |
| --- | --- | --- |
| Physical health (Before COVID-19) | 1: terrible, 2: poor, 3: average, 4: good, and 5: excellent | Survey |
| Physical health (After COVID-19) | 1: terrible, 2: poor, 3: average, 4: good, and 5: excellent | Survey |
| Change in physical health | Change = After – Before (from -4 to 4) | Calculated |
| 24-hour cycle (Before COVID-19) | 1: terrible, 2: poor, 3: average, 4: good, and 5: excellent | Survey |
| 24-hour cycle (After COVID-19) | 1: terrible, 2: poor, 3: average, 4: good, and 5: excellent | Survey |
| Change in 24-hour cycle | Change = After – Before (from -4 to 4) | Calculated |
| Drowsiness (Before COVID-19) | 1: terrible, 2: poor, 3: average, 4: good, and 5: excellent | Survey |
| Drowsiness (After COVID-19) | 1: terrible, 2: poor, 3: average, 4: good, and 5: excellent | Survey |
| Change in drowsiness | Change = After – Before (from -4 to 4) | Calculated |
| Mental health (Before COVID-19) | 1: terrible, 2: poor, 3: average, 4: good, and 5: excellent | Survey |
| Mental health (After COVID-19) | 1: terrible, 2: poor, 3: average, 4: good, and 5: excellent | Survey |
| Change in mental health | Change = After – Before (from -4 to 4) | Calculated |
| Sleep quality (Before COVID-19) | 1: terrible, 2: poor, 3: average, 4: good, and 5: excellent | Survey |
| Sleep quality (After COVID-19) | 1: terrible, 2: poor, 3: average, 4: good, and 5: excellent | Survey |
| Change in sleep quality | Change = After – Before (from -4 to 4) | Calculated |
| Stress (Before COVID-19) | 1: terrible, 2: poor, 3: average, 4: good, and 5: excellent | Survey |
| Stress (After COVID-19) | 1: terrible, 2: poor, 3: average, 4: good, and 5: excellent | Survey |
| Change in stress | Change = After – Before (from -4 to 4) | Calculated |
| Social well-being (Before COVID-19) | 1: terrible, 2: poor, 3: average, 4: good, and 5: excellent | Survey |
| Social well-being (After COVID-19) | 1: terrible, 2: poor, 3: average, 4: good, and 5: excellent | Survey |
| Change in social well-being | Change = After – Before (from -4 to 4) | Calculated |
| Work-life balance (Before COVID-19) | 1: terrible, 2: poor, 3: average, 4: good, and 5: excellent | Survey |
| Work-life balance (After COVID-19) | 1: terrible, 2: poor, 3: average, 4: good, and 5: excellent | Survey |
| Change in work-life balance | Change = After – Before (from -4 to 4) | Calculated |
| Productivity (Before COVID-19) | 1: terrible, 2: poor, 3: average, 4: good, and 5: excellent | Survey |
| Productivity (After COVID-19) | 1: terrible, 2: poor, 3: average, 4: good, and 5: excellent | Survey |
| Change in productivity | Change = After – Before (from -4 to 4) | Calculated |
| Job satisfaction (Before COVID-19) | 1: terrible, 2: poor, 3: average, 4: good, and 5: excellent | Survey |
| Job satisfaction (After COVID-19) | 1: terrible, 2: poor, 3: average, 4: good, and 5: excellent | Survey |
| Change in job satisfaction | Change = After – Before (from -4 to 4) | Calculated |
| Work engagement (Before COVID-19) | 1: terrible, 2: poor, 3: average, 4: good, and 5: excellent | Survey |
| Work engagement (After COVID-19) | 1: terrible, 2: poor, 3: average, 4: good, and 5: excellent | Survey |
| Change in work engagement | Change = After – Before (from -4 to 4) | Calculated |
| Work enjoyment (Before COVID-19) | 1: terrible, 2: poor, 3: average, 4: good, and 5: excellent | Survey |
| Work enjoyment (After COVID-19) | 1: terrible, 2: poor, 3: average, 4: good, and 5: excellent | Survey |
| Change in work enjoyment | Change = After – Before (from -4 to 4) | Calculated |
| Energy (Before COVID-19) | 1: terrible, 2: poor, 3: average, 4: good, and 5: excellent | Survey |
| Energy (After COVID-19) | 1: terrible, 2: poor, 3: average, 4: good, and 5: excellent | Survey |
| Change in energy | Change = After – Before (from -4 to 4) | Calculated |
| Concentration (Before COVID-19) | 1: terrible, 2: poor, 3: average, 4: good, and 5: excellent | Survey |
| Concentration (After COVID-19) | 1: terrible, 2: poor, 3: average, 4: good, and 5: excellent | Survey |
| Change in concentration | Change = After – Before (from -4 to 4) | Calculated |

S1.2 Table. Predictive Variables

| **Type of variables** | **Coding** |
| --- | --- |
| Satisfaction with WFH space | 1: extremely dissatisfied, 2: somewhat dissatisfied, 3: neither satisfied nor dissatisfied, 4: somewhat satisfied, 5: extremely satisfied |
| Temperature^*^ | 1: extremely dissatisfied, 2: somewhat dissatisfied, 3: neither satisfied nor dissatisfied, 4: somewhat satisfied, 5: extremely satisfied |
| Lighting^*^ | 1: extremely dissatisfied, 2: somewhat dissatisfied, 3: neither satisfied nor dissatisfied, 4: somewhat satisfied, 5: extremely satisfied |
| Noise outside^*^ | 1: extremely dissatisfied, 2: somewhat dissatisfied, 3: neither satisfied nor dissatisfied, 4: somewhat satisfied, 5: extremely satisfied |
| Noise inside^*^ | 1: extremely dissatisfied, 2: somewhat dissatisfied, 3: neither satisfied nor dissatisfied, 4: somewhat satisfied, 5: extremely satisfied |
| Aesthetical pleasure^*^ | 1: extremely dissatisfied, 2: somewhat dissatisfied, 3: neither satisfied nor dissatisfied, 4: somewhat satisfied, 5: extremely satisfied |
| Ergonomic comfort^*^ | 1: extremely dissatisfied, 2: somewhat dissatisfied, 3: neither satisfied nor dissatisfied, 4: somewhat satisfied, 5: extremely satisfied |
| Physiological comfort^*^ | 1: extremely dissatisfied, 2: somewhat dissatisfied, 3: neither satisfied nor dissatisfied, 4: somewhat satisfied, 5: extremely satisfied |
| Concentration^*^ | 1: extremely dissatisfied, 2: somewhat dissatisfied, 3: neither satisfied nor dissatisfied, 4: somewhat satisfied, 5: extremely satisfied |
| Relieving stress^*^ | 1: extremely dissatisfied, 2: somewhat dissatisfied, 3: neither satisfied nor dissatisfied, 4: somewhat satisfied, 5: extremely satisfied |
| Attachment to home^*^ | 1: extremely dissatisfied, 2: somewhat dissatisfied, 3: neither satisfied nor dissatisfied, 4: somewhat satisfied, 5: extremely satisfied |
| Privacy^*^ | 1: extremely dissatisfied, 2: somewhat dissatisfied, 3: neither satisfied nor dissatisfied, 4: somewhat satisfied, 5: extremely satisfied |
| Living alone | 0: living with others, 1: living alone |
| Living with children | 0: no children, 1: living with child(ren) |
| Housing type | 0: house, 1: apartment |
| Attachment to neighbor | 1: not at all, 2: a little, 3: a moderate amount, 4: a lot, 5: a great deal |
| Prior experience of WFH | 0: yes, 1: no |
| WFH frequency | 1: less than 1 day a week, 2: 1-2 days a week, 3: 3-4 days a week, 4: more than 5 days a week |
| WFH compulsoriness | 0: compulsory, 1: voluntary |
| Job insecurity | 0: other, 1: full-time |
| Job position | 0: subordinate, 1: superior |
| Interaction required | 1: not at all, 2: a little, 3: a moderate amount, 4: a lot, 5: a great deal |
| Age | 1: under 21, 2: 21-25, 3: 26-30, 4: 31-35, 5: 36-40, 6: 41-45, 7: 46-50, 8: 51-55, 9: 56-60, 10: 61-65, 11: 66-70, 12: 71 or older |
| Sex | 0: male, 1: female |
| Marriage | 0: not married, 1: married |

* This indicates satisfaction with WFH space in terms of that variable.
