# Supplementary material for: How are people coping with working from home during the COVID-19 pandemic?: Experiences from the Netherlands and South Korea
Source: PLoS One. 2024 Apr 18;19(4):e0301351. doi: 10.1371/journal.pone.0301351 (PMC11025775; doi:10.1371/journal.pone.0301351)
Supplement: S2 Appendix — (DOCX) [file pone.0301351.s002.docx]

**Supporting information**

**S2 Appendix: Regression analyses**

S2.1 Table. Regression models for KR participants (n=195)

| **DV** | **IV** | ***Adj. R^2^*** | **Durbin-Watson** | **b** | **SE** | ***β*** | **t** | ***p*** | **95% CI** | |
| --- | --- | --- | --- | --- | --- | --- | --- | --- | --- | --- |
|  |  |  |  |  |  |  |  |  | **LB** | **UB** |
| Physical health |  | 0.193*** | 1.870 |  |  |  |  |  |  |  |
|  | S_Noise outside |  |  | 0.287 | 0.070 | 0.286 | 4.072 | <.001 | 0.148 | 0.425 |
|  | WFH days per week |  |  | -0.201 | 0.057 | -0.229 | -3.545 | <.001 | -0.313 | -0.089 |
|  | S_Relieving stress |  |  | 0.210 | 0.061 | 0.243 | 3.435 | <.001 | 0.089 | 0.330 |
|  | S_Lighting |  |  | -0.183 | 0.058 | -0.219 | -3.163 | 0.002 | -0.297 | -0.069 |
| 24-hour cycle |  | 0.221*** | 1.967 |  |  |  |  |  |  |  |
|  | S_Relieving stress |  |  | 0.366 | 0.066 | 0.377 | 5.567 | <.001 | 0.236 | 0.496 |
|  | Housing (0: house, 1: apartment) |  |  | -1.739 | 0.507 | -0.222 | -3.433 | <.001 | -2.739 | -0.740 |
|  | WFH days per week |  |  | -0.228 | 0.064 | -0.231 | -3.588 | <.001 | -0.353 | -0.103 |
|  | S_Temperature |  |  | -0.161 | 0.072 | -0.152 | -2.221 | 0.028 | -0.304 | -0.018 |
| Drowsiness |  | 0.212*** | 2.128 |  |  |  |  |  |  |  |
|  | S_Concentration |  |  | 0.207 | 0.057 | 0.247 | 3.594 | <.001 | 0.093 | 0.320 |
|  | S_Relieving stress |  |  | 0.207 | 0.057 | 0.247 | 3.626 | <.001 | 0.094 | 0.320 |
|  | Employment (0: other, 1: full-time) |  |  | 0.530 | 0.177 | 0.193 | 2.996 | 0.003 | 0.181 | 0.879 |
| Mental health |  | 0.149*** | 1.871 |  |  |  |  |  |  |  |
|  | S_Relieving stress |  |  | 0.333 | 0.058 | 0.379 | 5.711 | <.001 | 0.218 | 0.448 |
|  | Housing (0: house, 1: apartment) |  |  | -0.946 | 0.470 | -0.133 | -2.011 | 0.046 | -1.874 | -0.018 |
| Sleep quality |  | 0.171*** | 1.955 |  |  |  |  |  |  |  |
|  | S_Relieving stress |  |  | 0.384 | 0.076 | 0.336 | 5.087 | <.001 | 0.235 | 0.534 |
|  | Age |  |  | -0.187 | 0.063 | -0.198 | -2.988 | 0.003 | -0.311 | -0.064 |
|  | WFH experience (0: yes, 1: no) |  |  | 0.371 | 0.185 | 0.133 | 2.003 | 0.047 | 0.006 | 0.736 |
| Depression |  | 0.162*** | 1.909 |  |  |  |  |  |  |  |
|  | S_Relieving stress |  |  | 0.317 | 0.054 | 0.388 | 5.907 | <.001 | 0.211 | 0.423 |
|  | WFH days per week |  |  | -0.130 | 0.055 | -0.156 | -2.378 | 0.018 | -0.237 | -0.022 |
| Stress |  | 0.131*** | 1.732 |  |  |  |  |  |  |  |
|  | S_Relieving stress |  |  | 0.414 | 0.075 | 0.369 | 5.508 | <.001 | 0.266 | 0.562 |
| Social well-being |  | 0.093*** | 1.948 |  |  |  |  |  |  |  |
|  | S_Relieving stress |  |  | 0.261 | 0.073 | 0.245 | 3.558 | <.001 | 0.116 | 0.405 |
|  | WFH days per week |  |  | -0.212 | 0.074 | -0.196 | -2.858 | 0.005 | -0.358 | -0.066 |
|  | Attachment to neighbor |  |  | -0.158 | 0.072 | -0.151 | -2.189 | 0.030 | -0.300 | -0.016 |
| Work-life balance |  | 0.190*** | 1.806 |  |  |  |  |  |  |  |
|  | S_Relieving stress |  |  | 0.528 | 0.087 | 0.392 | 6.048 | <.001 | 0.356 | 0.700 |
|  | Housing (0: house, 1: apartment) |  |  | -1.727 | 0.701 | -0.159 | -2.462 | 0.015 | -3.110 | -0.343 |
|  | Age |  |  | -0.149 | 0.072 | -0.134 | -2.069 | 0.040 | -0.291 | -0.007 |
| Productivity |  | 0.199*** | 1.719 |  |  |  |  |  |  |  |
|  | S_Concentration |  |  | 0.292 | 0.066 | 0.303 | 4.411 | <.001 | 0.161 | 0.422 |
|  | S_Relieving stress |  |  | 0.242 | 0.072 | 0.251 | 3.348 | <.001 | 0.100 | 0.385 |
|  | Children (0: no, 1: yes) |  |  | -0.325 | 0.125 | -0.168 | -2.594 | 0.010 | -0.571 | -0.078 |
|  | S_Privacy |  |  | -0.148 | 0.070 | -0.150 | -2.097 | 0.037 | -0.287 | -0.009 |
| Job satisfaction |  | 0.082*** | 1.814 |  |  |  |  |  |  |  |
|  | S_Relieving stress |  |  | 0.201 | 0.060 | 0.230 | 3.332 | 0.001 | 0.082 | 0.320 |
|  | Age |  |  | -0.128 | 0.050 | -0.177 | -2.560 | 0.011 | -0.226 | -0.029 |
| Work engagement |  | 0.082*** | 1.649 |  |  |  |  |  |  |  |
|  | Housing (0: house, 1: apartment) |  |  | -2.214 | 0.517 | -0.295 | -4.283 | <.001 | -3.233 | -1.194 |
| Work enjoyment |  | 0.124*** | 1.836 |  |  |  |  |  |  |  |
|  | S_Relieving stress |  |  | 0.214 | 0.055 | 0.266 | 3.921 | <.001 | 0.106 | 0.322 |
|  | Age |  |  | -0.131 | 0.045 | -0.198 | -2.895 | 0.004 | -0.220 | -0.042 |
|  | WFH experience (0: yes, 1: no) |  |  | 0.269 | 0.134 | 0.137 | 2.007 | 0.046 | 0.005 | 0.533 |
| Energy |  | 0.227*** | 1.765 |  |  |  |  |  |  |  |
|  | S_Relieving stress |  |  | 0.431 | 0.067 | 0.409 | 6.445 | <.001 | 0.299 | 0.563 |
|  | Housing (0: house, 1: apartment) |  |  | -1.626 | 0.538 | -0.191 | -3.024 | 0.003 | -2.686 | -0.565 |
|  | Age |  |  | -0.126 | 0.055 | -0.145 | -2.283 | 0.024 | -0.236 | -0.017 |
|  | Interaction required while working |  |  | -0.165 | 0.075 | -0.139 | -2.185 | 0.030 | -0.313 | -0.016 |
| Concentration |  | 0.230*** | 1.702 |  |  |  |  |  |  |  |
|  | S_Concentration |  |  | 0.317 | 0.072 | 0.304 | 4.416 | <.001 | 0.175 | 0.458 |
|  | Interaction required while working |  |  | -0.240 | 0.075 | -0.205 | -3.200 | 0.002 | -0.388 | -0.092 |
|  | Housing (0: house, 1: apartment) |  |  | -1.568 | 0.534 | -0.186 | -2.936 | 0.004 | -2.621 | -0.515 |
|  | S_Relieving stress |  |  | 0.272 | 0.076 | 0.260 | 3.593 | <.001 | 0.122 | 0.421 |
|  | S_Attachment to home |  |  | -0.228 | 0.083 | -0.189 | -2.750 | 0.007 | -0.391 | -0.064 |
|  | Marriage (0: not married, 1: married) |  |  | 0.274 | 0.135 | 0.130 | 2.024 | 0.044 | 0.007 | 0.540 |

*** *p* < .001

S2.2 Table. Regression models for NL participants (n=112)

| **DV** | **IV** | ***Adj. R^2^*** | **Durbin-Watson** | **b** | **SE** | ***β*** | **t** | ***p*** | **95% CI** | |
| --- | --- | --- | --- | --- | --- | --- | --- | --- | --- | --- |
|  |  |  |  |  |  |  |  |  | **LB** | **UB** |
| Physical health |  | 0.141*** | 1.888 |  |  |  |  |  |  |  |
|  | Attachment to neighbor |  |  | 0.230 | 0.066 | 0.329 | 3.496 | <.001 | 0.100 | 0.360 |
|  | Role (0: subordinate, 1: superior) |  |  | 0.345 | 0.154 | 0.202 | 2.248 | 0.027 | 0.041 | 0.650 |
|  | Living alone (0: no, 1: yes) |  |  | 0.512 | 0.256 | 0.189 | 2.001 | 0.048 | 0.005 | 1.020 |
| 24-hour cycle |  | 0.027* | 2.211 |  |  |  |  |  |  |  |
|  | S_Relieving stress |  |  | 0.161 | 0.080 | 0.189 | 2.005 | 0.047 | 0.002 | 0.320 |
| Drowsiness |  | 0.162*** | 1.971 |  |  |  |  |  |  |  |
|  | S_Ergonomic comfort |  |  | 0.233 | 0.079 | 0.272 | 2.939 | 0.004 | 0.076 | 0.390 |
|  | S_Aesthetical pleasure |  |  | 0.187 | 0.071 | 0.245 | 2.642 | 0.009 | 0.047 | 0.328 |
| Mental health |  | 0.153*** | 2.228 |  |  |  |  |  |  |  |
|  | S_Relieving stress |  |  | 0.245 | 0.088 | 0.261 | 2.780 | 0.006 | 0.070 | 0.419 |
|  | S_Ergonomic comfort |  |  | 0.249 | 0.097 | 0.241 | 2.568 | 0.012 | 0.057 | 0.441 |
| Sleep quality |  | 0.136*** | 2.110 |  |  |  |  |  |  |  |
|  | S_Noise outside |  |  | 0.367 | 0.116 | 0.281 | 3.154 | 0.002 | 0.136 | 0.597 |
|  | Satisfaction with WFH space |  |  | 0.259 | 0.086 | 0.269 | 3.018 | 0.003 | 0.089 | 0.430 |
| Depression |  | 0.185*** | 2.117 |  |  |  |  |  |  |  |
|  | S_Concentration |  |  | 0.178 | 0.066 | 0.250 | 2.674 | 0.009 | 0.046 | 0.309 |
|  | WFH experience (0: yes, 1: no) |  |  | -0.379 | 0.149 | -0.223 | -2.543 | 0.012 | -0.674 | -0.083 |
|  | Satisfaction with WFH space |  |  | 0.164 | 0.071 | 0.213 | 2.295 | 0.024 | 0.022 | 0.305 |
| Stress |  | 0.098** | 2.269 |  |  |  |  |  |  |  |
|  | S_Ergonomic comfort |  |  | 0.252 | 0.090 | 0.256 | 2.803 | 0.006 | 0.074 | 0.431 |
|  | S_Lighting |  |  | 0.176 | 0.082 | 0.198 | 2.161 | 0.033 | 0.015 | 0.338 |
| Social well-being |  | 0.226*** | 2.455 |  |  |  |  |  |  |  |
|  | Attachment to neighbor |  |  | 0.298 | 0.081 | 0.319 | 3.684 | <.001 | 0.137 | 0.458 |
|  | S_Aesthetical pleasure |  |  | 0.251 | 0.084 | 0.258 | 2.976 | 0.004 | 0.084 | 0.417 |
|  | WFH compulsoriness (0: yes, 1: no) |  |  | 0.821 | 0.331 | 0.209 | 2.479 | 0.015 | 0.164 | 1.478 |
| Work-life balance |  | 0.233*** | 2.016 |  |  |  |  |  |  |  |
|  | S_Noise inside |  |  | 0.436 | 0.126 | 0.295 | 3.463 | <.001 | 0.186 | 0.686 |
|  | Satisfaction with WFH space |  |  | 0.346 | 0.089 | 0.326 | 3.886 | <.001 | 0.170 | 0.523 |
|  | Role (0: subordinate, 1: superior) |  |  | 0.462 | 0.199 | 0.198 | 2.321 | 0.022 | 0.067 | 0.857 |
| Productivity |  | 0.124*** | 1.987 |  |  |  |  |  |  |  |
|  | S_Concentration |  |  | 0.318 | 0.078 | 0.364 | 4.058 | <.001 | 0.163 | 0.473 |
| Job satisfaction |  | 0.147*** | 1.845 | -1.692 | 0.303 |  | -5.578 | <.001 | -2.293 | -1.091 |
|  | Satisfaction with WFH space |  |  | 0.237 | 0.083 | 0.273 | 2.872 | 0.005 | 0.074 | 0.401 |
|  | S_Relieving stress |  |  | 0.178 | 0.079 | 0.214 | 2.258 | 0.026 | 0.022 | 0.334 |
| Work engagement |  | 0.263*** | 1.997 |  |  |  |  |  |  |  |
|  | S_Concentration |  |  | 0.231 | 0.067 | 0.314 | 3.469 | <.001 | 0.099 | 0.364 |
|  | S_Temperature |  |  | -0.272 | 0.064 | -0.360 | -4.261 | <.001 | -0.399 | -0.145 |
|  | S_Ergonomic comfort |  |  | 0.220 | 0.075 | 0.263 | 2.936 | 0.004 | 0.071 | 0.368 |
|  | Employment (0: other, 1: full-time) |  |  | 0.316 | 0.151 | 0.176 | 2.088 | 0.039 | 0.016 | 0.616 |
| Work enjoyment |  | 0.162*** | 1.995 |  |  |  |  |  |  |  |
|  | S_Relieving stress |  |  | 0.244 | 0.084 | 0.280 | 2.917 | 0.004 | 0.078 | 0.411 |
|  | S_Concentration |  |  | 0.185 | 0.080 | 0.222 | 2.307 | 0.023 | 0.026 | 0.344 |
| Energy |  | 0.355*** | 2.148 |  |  |  |  |  |  |  |
|  | S_Concentration |  |  | 0.170 | 0.073 | 0.202 | 2.314 | 0.023 | 0.024 | 0.315 |
|  | WFH experience (0: yes, 1: no) |  |  | -0.641 | 0.157 | -0.319 | -4.088 | <.001 | -0.951 | -0.330 |
|  | Satisfaction with WFH space |  |  | 0.223 | 0.078 | 0.246 | 2.876 | 0.005 | 0.069 | 0.377 |
|  | S_Relieving stress |  |  | 0.190 | 0.075 | 0.220 | 2.534 | 0.013 | 0.041 | 0.339 |
| Concentration |  | 0.270*** | 2.286 |  |  |  |  |  |  |  |
|  | S_Concentration |  |  | 0.346 | 0.081 | 0.373 | 4.256 | <.001 | 0.185 | 0.507 |
|  | S_Noise outside |  |  | 0.326 | 0.112 | 0.240 | 2.925 | 0.004 | 0.105 | 0.548 |
|  | Satisfaction with WFH space |  |  | 0.177 | 0.088 | 0.176 | 2.015 | 0.046 | 0.003 | 0.351 |

**p*<0.05, ***p*<0.01, ****p*<0.001
