# Supplementary material for: How are people coping with working from home during the COVID-19 pandemic?: Experiences from the Netherlands and South Korea
Source: PLoS One. 2024 Apr 18;19(4):e0301351. doi: 10.1371/journal.pone.0301351 (PMC11025775; doi:10.1371/journal.pone.0301351)
Supplement: S3 Appendix — (DOCX) [file pone.0301351.s003.docx]

**Supporting information**

**S3 Appendix: Independent-samples t-tests**

S3.1 Table. Comparison between NL respondents and KR respondents

|  | Mean | | Std. Deviation | | *t* | *df* | *p* |
| --- | --- | --- | --- | --- | --- | --- | --- |
|  | NL (n=112) | KR (n=195) | NL (n=112) | KR (n=195) |  |  |  |
| Physical health | -0.420 | -0.180 | 0.845 | 0.860 | -2.331 | 234.7 | 0.021 |
| 24-hour cycle | -0.180 | -0.040 | 0.970 | 0.965 | -1.244 | 305.0 | 0.214 |
| Drowsiness | -0.440 | 0.020 | 0.888 | 0.834 | -4.397 | 219.7 | <.001 |
| Mental health | -0.630 | 0.160 | 1.074 | 0.876 | -6.690 | 195.7 | <.001 |
| Sleep quality | -0.280 | 0.360 | 1.050 | 1.137 | -4.847 | 305.0 | <.001 |
| Depression | -0.490 | 0.000 | 0.838 | 0.812 | -4.997 | 225.5 | <.001 |
| Stress | -0.370 | 0.340 | 1.022 | 1.117 | -5.525 | 305.0 | <.001 |
| Social well-being | -0.900 | -0.560 | 1.131 | 1.060 | -2.661 | 305.0 | 0.008 |
| Work-life balance | -0.480 | 0.420 | 1.162 | 1.338 | -5.928 | 305.0 | <.001 |
| Productivity | -0.460 | -0.110 | 1.030 | 0.960 | -2.992 | 218.2 | 0.003 |
| Job satisfaction | -0.360 | 0.230 | 0.948 | 0.869 | -5.390 | 215.4 | <.001 |
| Work engagement | -0.530 | -0.180 | 0.870 | 0.927 | -3.231 | 305.0 | 0.001 |
| Work enjoyment | -0.450 | 0.070 | 0.988 | 0.800 | -4.704 | 192.2 | <.001 |
| Energy | -0.830 | 0.100 | 0.994 | 1.048 | -7.605 | 305.0 | <.001 |
| Concentration | -0.710 | -0.140 | 1.096 | 1.039 | -4.512 | 305.0 | <.001 |

S3.2 Table. Comparison between those living in houses and in apartments in terms of physical/mental health and productivity changes

|  | Mean | | Std. Deviation | | *t* | *df* | *p* |
| --- | --- | --- | --- | --- | --- | --- | --- |
|  | House  (n=61) | Apartment (n=51) | House  (n=61) | Apartment (n=51) |  |  |  |
| Physical health | -0.480 | -0.350 | 0.906 | 0.770 | -0.762 | 110.0 | 0.448 |
| 24-hour cycle | -0.160 | -0.200 | 1.067 | 0.849 | 0.174 | 110.0 | 0.862 |
| Drowsiness | -0.480 | -0.390 | 0.942 | 0.827 | -0.492 | 110.0 | 0.623 |
| Mental health | -0.460 | -0.840 | 1.089 | 1.027 | 1.908 | 110.0 | 0.059 |
| Sleep quality | -0.210 | -0.350 | 1.018 | 1.092 | 0.700 | 110.0 | 0.485 |
| Depression | -0.410 | -0.590 | 0.824 | 0.853 | 1.123 | 110.0 | 0.264 |
| Stress | -0.280 | -0.470 | 1.051 | 0.987 | 0.989 | 110.0 | 0.325 |
| Social well-being | -0.750 | -1.080 | 1.120 | 1.129 | 1.521 | 110.0 | 0.131 |
| Work-life balance | -0.570 | -0.370 | 1.117 | 1.216 | -0.912 | 110.0 | 0.364 |
| Productivity | -0.380 | -0.570 | 1.067 | 0.985 | 0.980 | 110.0 | 0.329 |
| Job satisfaction | -0.280 | -0.450 | 0.819 | 1.083 | 0.958 | 110.0 | 0.340 |
| Work engagement | -0.480 | -0.590 | 0.788 | 0.963 | 0.682 | 110.0 | 0.497 |
| Work enjoyment | -0.400 | -0.510 | 0.978 | 1.007 | 0.581 | 109.0 | 0.562 |
| Energy | -0.660 | -1.040 | 0.929 | 1.038 | 2.062 | 110.0 | 0.042 |
| Concentration | -0.490 | -0.960 | 0.942 | 1.216 | 2.299 | 110.0 | 0.023 |

S3.3 Table. Comparison between those living in houses and in apartments in terms of satisfaction with WFH environment

|  | Mean | | Std. Deviation | | *t* | *df* | *p* |
| --- | --- | --- | --- | --- | --- | --- | --- |
|  | House  (n=61) | Apartment (n=51) | House  (n=61) | Apartment (n=51) |  |  |  |
| Satisfaction with WFH space | 3.340 | 3.250 | 1.078 | 1.111 | 0.431 | 110.0 | 0.667 |
| Temperature | 3.660 | 3.550 | 1.182 | 1.119 | 0.488 | 110.0 | 0.627 |
| Lighting | 3.570 | 3.350 | 1.132 | 1.163 | 1.015 | 110.0 | 0.312 |
| Noise inside | 3.850 | 3.980 | 0.910 | 0.735 | -0.808 | 110.0 | 0.421 |
| Noise outside | 3.930 | 3.710 | 0.793 | 0.855 | 1.465 | 110.0 | 0.146 |
| Aesthetical pleasure | 3.490 | 3.180 | 1.135 | 1.178 | 1.439 | 110.0 | 0.153 |
| Concentration | 3.330 | 2.710 | 1.221 | 1.045 | 2.865 | 110.0 | 0.005 |
| Ergonomic comfort | 3.740 | 3.250 | 0.929 | 1.111 | 2.466 | 97.7 | 0.015 |
| Physiological comfort | 3.610 | 3.250 | 0.936 | 1.129 | 1.773 | 97.2 | 0.079 |
| Relieving stress | 3.230 | 2.880 | 1.160 | 1.107 | 1.610 | 110.0 | 0.110 |
| Attachment to home | 4.000 | 3.350 | 0.816 | 1.092 | 3.493 | 91.1 | <.001 |
| Attachment to neighbor | 3.330 | 2.780 | 1.165 | 1.205 | 2.421 | 110.0 | 0.017 |
| Privacy | 3.920 | 3.590 | 0.759 | 0.920 | 2.043 | 96.9 | 0.044 |

S3.4 Table. Comparison between those with and without prior WFH experience (KR respondents only)

|  | Mean | | Std. Deviation | | *t* | *df* | *p* |
| --- | --- | --- | --- | --- | --- | --- | --- |
|  | KR_With  (n=41) | KR_Without (n=154) | KR_With  (n=41) | KR_Without (n=154) |  |  |  |
| Physical health | -0.410 | -0.120 | 0.921 | 0.835 | -1.942 | 193.0 | 0.054 |
| 24-hour cycle | -0.370 | 0.050 | 1.019 | 0.934 | -2.374 | 59.1 | 0.021 |
| Drowsiness | -0.070 | 0.040 | 0.685 | 0.870 | -0.764 | 193.0 | 0.446 |
| Mental health | -0.100 | 0.230 | 0.831 | 0.877 | -2.174 | 193.0 | 0.031 |
| Sleep quality | 0.050 | 0.440 | 1.071 | 1.143 | -1.980 | 193.0 | 0.049 |
| Depression | -0.290 | 0.080 | 0.642 | 0.837 | -2.636 | 193.0 | 0.009 |
| Stress | 0.020 | 0.430 | 0.908 | 1.154 | -2.384 | 78.0 | 0.020 |
| Social well-being | -0.780 | -0.500 | 1.037 | 1.062 | -1.510 | 193.0 | 0.133 |
| Work-life balance | 0.070 | 0.510 | 1.385 | 1.315 | -1.854 | 193.0 | 0.065 |
| Productivity | -0.120 | -0.100 | 0.900 | 0.978 | -0.107 | 193.0 | 0.915 |
| Job satisfaction | 0.150 | 0.250 | 0.853 | 0.875 | -0.699 | 193.0 | 0.485 |
| Work engagement | -0.290 | -0.150 | 1.006 | 0.906 | -0.879 | 193.0 | 0.380 |
| Work enjoyment | -0.150 | 0.120 | 0.727 | 0.811 | -1.932 | 193.0 | 0.055 |
| Energy | -0.150 | 0.160 | 0.853 | 1.087 | -1.684 | 193.0 | 0.094 |
| Concentration | -0.100 | -0.150 | 1.114 | 1.021 | 0.283 | 193.0 | 0.777 |

S3.5 Table. Comparison between those with and without prior WFH experience (NL respondents only)

|  | Mean | | Std. Deviation | | *t* | *df* | *p* |
| --- | --- | --- | --- | --- | --- | --- | --- |
|  | NL_With  (n=48) | NL_Without (n=64) | NL_With  (n=48) | NL_Without (n=64) |  |  |  |
| Physical health | -0.290 | -0.520 | 0.824 | 0.854 | 1.394 | 110.0 | 0.166 |
| 24-hour cycle | -0.100 | -0.230 | 0.831 | 1.065 | 0.726 | 109.8 | 0.469 |
| Drowsiness | -0.350 | -0.500 | 0.838 | 0.926 | 0.859 | 110.0 | 0.392 |
| Mental health | -0.400 | -0.810 | 1.086 | 1.037 | 2.062 | 110.0 | 0.042 |
| Sleep quality | -0.400 | -0.190 | 1.067 | 1.037 | -1.039 | 110.0 | 0.301 |
| Depression | -0.250 | -0.670 | 0.786 | 0.837 | 2.710 | 110.0 | 0.008 |
| Stress | -0.310 | -0.410 | 1.035 | 1.019 | 0.479 | 110.0 | 0.633 |
| Social well-being | -0.750 | -1.020 | 1.101 | 1.148 | 1.233 | 110.0 | 0.220 |
| Work-life balance | -0.500 | -0.470 | 1.272 | 1.083 | -0.140 | 110.0 | 0.889 |
| Productivity | -0.210 | -0.660 | 1.051 | 0.979 | 2.321 | 110.0 | 0.022 |
| Job satisfaction | -0.230 | -0.450 | 0.951 | 0.942 | 1.241 | 110.0 | 0.217 |
| Work engagement | -0.310 | -0.690 | 0.657 | 0.974 | 2.430 | 108.9 | 0.017 |
| Work enjoyment | -0.290 | -0.570 | 0.967 | 0.995 | 1.485 | 109.0 | 0.140 |
| Energy | -0.440 | -1.120 | 0.848 | 1.000 | 3.838 | 110.0 | <.001 |
| Concentration | -0.560 | -0.810 | 1.090 | 1.097 | 1.197 | 110.0 | 0.234 |

S3.6 Table. Comparison according to the frequency of WFH (Compulsory group only)

|  | Mean | | Std. Deviation | | *t* | *df* | *p* |
| --- | --- | --- | --- | --- | --- | --- | --- |
|  | Compulsory 3 days or more (n=88) | Compulsory 2 days or less (n=83) | Compulsory 3 days or more (n=88) | Compulsory 2 days or less (n=83) |  |  |  |
| Physical health | -0.470 | -0.100 | 1.039 | 0.726 | -2.708 | 156.0 | 0.008 |
| 24-hour cycle | -0.360 | 0.130 | 0.985 | 0.880 | -3.478 | 168.5 | <.001 |
| Drowsiness | -0.170 | -0.040 | 1.031 | 0.917 | -0.901 | 168.4 | 0.369 |
| Mental health | -0.340 | 0.190 | 1.193 | 0.803 | -3.449 | 153.2 | <.001 |
| Sleep quality | 0.000 | 0.310 | 1.194 | 0.999 | -1.855 | 169.0 | 0.065 |
| Depression | -0.450 | 0.050 | 0.883 | 0.882 | -3.723 | 168.4 | <.001 |
| Stress | -0.180 | 0.370 | 1.120 | 1.134 | -3.221 | 169.0 | 0.002 |
| Social well-being | -0.900 | -0.550 | 1.062 | 1.107 | -2.071 | 169.0 | 0.040 |
| Work-life balance | -0.220 | 0.360 | 1.426 | 1.293 | -2.768 | 169.0 | 0.006 |
| Productivity | -0.260 | -0.350 | 1.088 | 0.876 | 0.581 | 169.0 | 0.562 |
| Job satisfaction | -0.070 | 0.130 | 0.968 | 0.960 | -1.360 | 169.0 | 0.176 |
| Work engagement | -0.370 | -0.250 | 0.986 | 0.948 | -0.824 | 169.0 | 0.411 |
| Work enjoyment | -0.240 | 0.020 | 1.072 | 0.765 | -1.853 | 157.5 | 0.066 |
| Energy | -0.440 | -0.020 | 1.276 | 1.137 | -2.270 | 168.5 | 0.024 |
| Concentration | -0.430 | -0.340 | 1.258 | 1.003 | -0.544 | 164.5 | 0.587 |

S3.7 Table. Comparison according to the frequency of WFH (Voluntary group only)

|  | Mean | | Std. Deviation | | *t* | *df* | *p* |
| --- | --- | --- | --- | --- | --- | --- | --- |
|  | Voluntary 3 days or more (n=68) | Voluntary 2 days or less (n=68) | Voluntary 3 days or more (n=68) | Voluntary 2 days or less (n=68) |  |  |  |
| Physical health | -0.350 | -0.150 | 0.748 | 0.815 | -1.534 | 134.0 | 0.127 |
| 24-hour cycle | -0.150 | 0.060 | 1.040 | 0.896 | -1.237 | 134.0 | 0.218 |
| Drowsiness | -0.250 | -0.160 | 0.780 | 0.704 | -0.692 | 134.0 | 0.490 |
| Mental health | -0.380 | 0.010 | 0.811 | 1.113 | -2.378 | 134.0 | 0.019 |
| Sleep quality | 0.160 | 0.030 | 1.205 | 1.184 | 0.646 | 134.0 | 0.519 |
| Depression | -0.340 | 0.060 | 0.745 | 0.751 | -3.095 | 134.0 | 0.002 |
| Stress | -0.120 | 0.280 | 1.030 | 1.157 | -2.114 | 134.0 | 0.036 |
| Social well-being | -0.940 | -0.310 | 1.091 | 1.026 | -3.482 | 134.0 | <.001 |
| Work-life balance | -0.100 | 0.340 | 1.283 | 1.277 | -2.010 | 134.0 | 0.046 |
| Productivity | -0.290 | -0.010 | 1.120 | 0.872 | -1.623 | 126.4 | 0.107 |
| Job satisfaction | -0.100 | 0.100 | 0.995 | 0.813 | -1.322 | 134.0 | 0.189 |
| Work engagement | -0.410 | -0.180 | 0.934 | 0.772 | -1.601 | 134.0 | 0.112 |
| Work enjoyment | -0.360 | 0.090 | 0.773 | 0.893 | -3.104 | 133.0 | 0.002 |
| Energy | -0.440 | -0.040 | 0.998 | 0.921 | -2.410 | 133.1 | 0.017 |
| Concentration | -0.380 | -0.210 | 1.120 | 0.939 | -0.995 | 130.1 | 0.321 |

S3.8 Table. Comparison according to the job position (KR respondents only)

|  | Mean | | Std. Deviation | | *t* | *df* | *p* |
| --- | --- | --- | --- | --- | --- | --- | --- |
|  | KR_ Subordinate (n=121) | KR_ Superior (n=74) | KR_ Subordinate (n=121) | KR_ Superior (n=74) |  |  |  |
| Physical health | -0.120 | -0.300 | 0.896 | 0.789 | 1.435 | 193.0 | 0.153 |
| 24-hour cycle | 0.060 | -0.190 | 0.934 | 1.002 | 1.743 | 193.0 | 0.083 |
| Drowsiness | 0.080 | -0.090 | 0.852 | 0.797 | 1.444 | 193.0 | 0.150 |
| Mental health | 0.220 | 0.070 | 0.944 | 0.746 | 1.275 | 180.6 | 0.204 |
| Sleep quality | 0.500 | 0.140 | 1.073 | 1.209 | 2.170 | 193.0 | 0.031 |
| Depression | 0.060 | -0.090 | 0.799 | 0.830 | 1.274 | 193.0 | 0.204 |
| Stress | 0.430 | 0.200 | 1.079 | 1.170 | 1.381 | 193.0 | 0.169 |
| Social well-being | -0.460 | -0.720 | 0.984 | 1.165 | 1.626 | 193.0 | 0.105 |
| Work-life balance | 0.520 | 0.240 | 1.205 | 1.524 | 1.408 | 193.0 | 0.161 |
| Productivity | -0.020 | -0.240 | 0.979 | 0.919 | 1.548 | 193.0 | 0.123 |
| Job satisfaction | 0.280 | 0.150 | 0.819 | 0.946 | 1.032 | 193.0 | 0.303 |
| Work engagement | -0.200 | -0.150 | 0.891 | 0.989 | -0.362 | 193.0 | 0.717 |
| Work enjoyment | 0.080 | 0.040 | 0.770 | 0.851 | 0.356 | 193.0 | 0.722 |
| Energy | 0.160 | 0.000 | 1.033 | 1.073 | 1.015 | 193.0 | 0.311 |
| Concentration | -0.080 | -0.230 | 1.053 | 1.014 | 0.959 | 193.0 | 0.339 |

S3.9 Table. Comparison according to the job position (NL respondents only)

|  | Mean | | Std. Deviation | | *t* | *df* | *p* |
| --- | --- | --- | --- | --- | --- | --- | --- |
|  | NL_ Subordinate (n=64) | NL_ Superior (n=48) | NL_ Subordinate (n=64) | NL_ Superior (n=48) |  |  |  |
| Physical health | -0.590 | -0.190 | 0.868 | 0.762 | -2.581 | 110.0 | 0.011 |
| 24-hour cycle | -0.220 | -0.120 | 1.046 | 0.866 | -0.505 | 110.0 | 0.615 |
| Drowsiness | -0.420 | -0.460 | 1.020 | 0.683 | 0.226 | 108.7 | 0.822 |
| Mental health | -0.700 | -0.540 | 1.217 | 0.849 | -0.786 | 110.0 | 0.433 |
| Sleep quality | -0.340 | -0.190 | 1.198 | 0.816 | -0.820 | 109.1 | 0.414 |
| Depression | -0.590 | -0.350 | 0.868 | 0.785 | -1.505 | 110.0 | 0.135 |
| Stress | -0.360 | -0.370 | 1.089 | 0.937 | 0.080 | 110.0 | 0.937 |
| Social well-being | -0.940 | -0.850 | 1.246 | 0.967 | -0.384 | 110.0 | 0.701 |
| Work-life balance | -0.700 | -0.190 | 1.268 | 0.938 | -2.474 | 110.0 | 0.015 |
| Productivity | -0.500 | -0.420 | 1.113 | 0.919 | -0.422 | 110.0 | 0.674 |
| Job satisfaction | -0.420 | -0.270 | 0.905 | 1.005 | -0.833 | 110.0 | 0.406 |
| Work engagement | -0.590 | -0.440 | 0.971 | 0.712 | -0.982 | 110.0 | 0.328 |
| Work enjoyment | -0.480 | -0.400 | 1.039 | 0.925 | -0.420 | 109.0 | 0.675 |
| Energy | -0.940 | -0.690 | 0.957 | 1.035 | -1.321 | 110.0 | 0.189 |
| Concentration | -0.800 | -0.580 | 1.057 | 1.145 | -1.021 | 110.0 | 0.310 |
