# Supplementary figures and images for: How are people coping with working from home during the COVID-19 pandemic?: Experiences from the Netherlands and South Korea
Source: PLoS One. 2024 Apr 18;19(4):e0301351. doi: 10.1371/journal.pone.0301351 (PMC11025775; doi:10.1371/journal.pone.0301351)

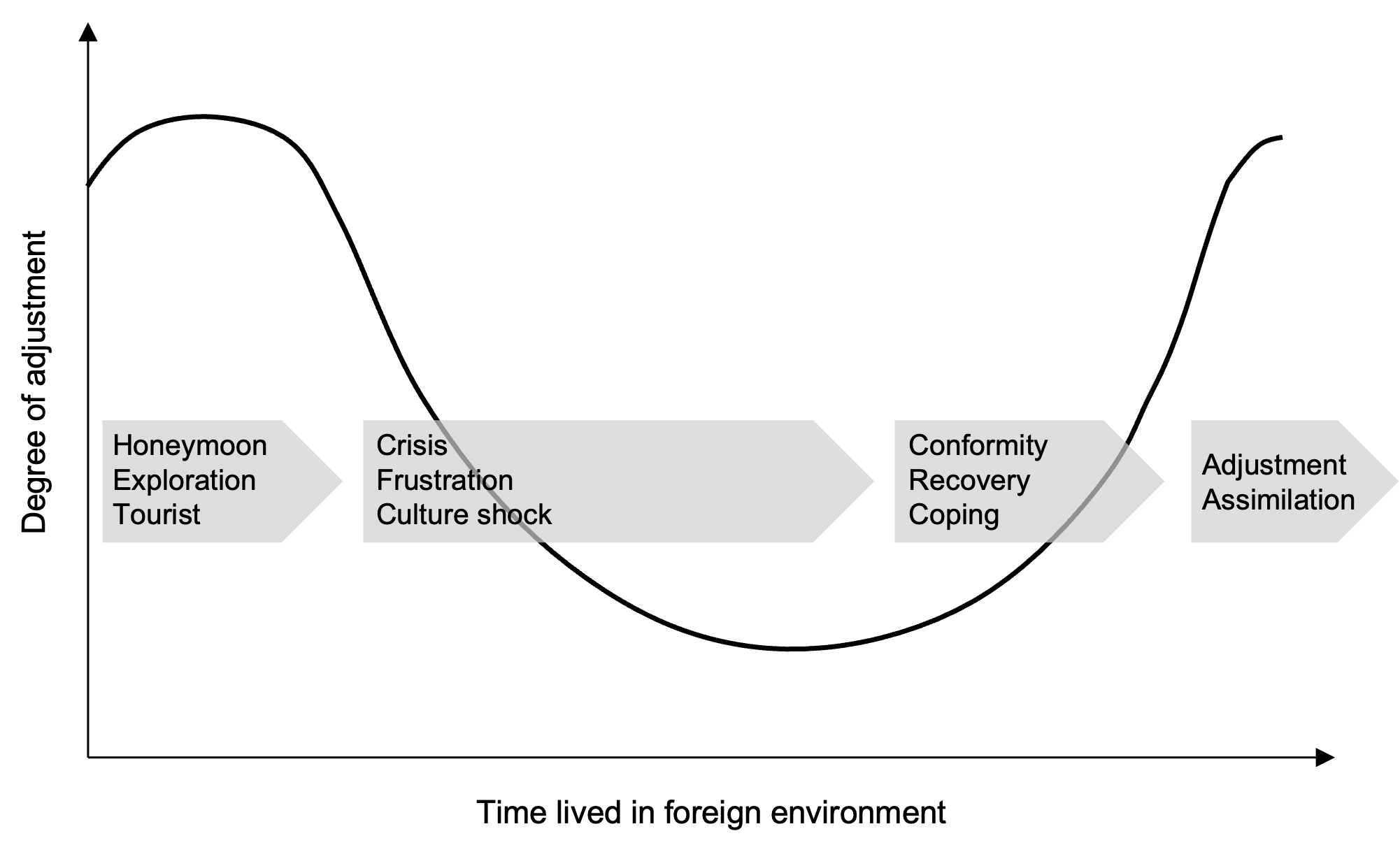

Supplement: S4 Appendix — Adapted from Lysgaard [36]. (TIF) [file pone.0301351.s004.tif]
